# Optimal range of lymph node dissection in patients with unilateral papillary thyroid carcinoma with lateral cervical lymph node metastasis

**DOI:** 10.3389/fonc.2023.1307937

**Published:** 2023-12-14

**Authors:** Liang Zhang, Zhaoming Ding, Jihua Han, Wen Bi, Chunlei Nie

**Affiliations:** Department of Head and Neck Surgery, Harbin Medical University Cancer Hospital, Harbin, China

**Keywords:** papillary thyroid carcinoma, lymph node metastasis, high-risk factors, recurrence-free survival, propensity score matching

## Abstract

**Background:**

Whether patients with unilateral papillary thyroid carcinoma (PTC) with lateral cervical lymph node metastasis (LLNM) require prophylactic central regional lymph node dissection (CLND) remains unclear. Herein, we investigated the independent risk factors associated with contralateral central lymph node metastasis (cCLNM) in unilateral PTC with LLNM and analyzed the optimal extent of lymph node dissection by comparing the 5-year recurrence-free survival rates.

**Materials and methods:**

We retrospectively analyzed 695 patients with unilateral papillary thyroid carcinoma and lateral cervical lymph node metastasis. Factors including sex, age, multifocal, location of primary tumor, tumor diameter, capsule invasion, thyroid nodular goiter, Hashimoto thyroiditis, ipsilateral central lymph node metastasis(iCLNM), and lateral cervical lymph node metastasis were analyzed using univariate and multivariate logistic regression analyses to explore the independent risk factors of cCLNM. Propensity scores were matched to compare the 5-year recurrence-free survival rates in patients divided by different lymph node metastases and dissections.

**Results:**

Of all patients who underwent bilateral (b)CLND, 52% (149/286) had cCLNM. Receiver operating characteristic (ROC) curve analysis was performed on 286 patients who underwent bCLND, for which a tumor diameter of 20.5 mm and number of LLNM of 3.5 were used as the thresholds for predicting cCLNM. The 5-year recurrence-free survival (RFS) rates in the cCLN-negative and cCLN-positive groups were 98.6% and 91.2%, with statistically significant differences (*P*=0.034). The 5-year RFS rates showed no significant difference between the ipsilateral (i)CLND and bCLND groups (*P*=0.235). Multifactorial regression analysis showed that tumor diameter >2 cm, presence of iCLNM, and number of LLNM >3 were independent risk factors of cCLNM.But male sex, young age (<45 years), multifocality, location of primary tumor, capsule invasion, thyroid nodular goiter, and Hashimoto thyroiditis were not associated with cCLNM.

**Conclusion:**

Not all unilateral PTC with LLNM require prophylactic cCLND; however, prophylactic cCLND is necessary in cases which display high-risk factors for cCLNM, including primary diameter >2 cm, iCLNM, and number of LLNM >3.

## 1 Introduction

Papillary thyroid carcinoma (PTC) is the most common malignant tumor of the head and neck, which first-choice treatment is surgery. The 2021 Chinese Society of Clinical Oncology (CSCO) guidelines for the diagnosis and treatment of differentiated thyroid cancer recommend that iCLND should be performed for patients with papillary thyroid cancer who are highly suspected or confirmed to have lymph node metastasis. While lateral cervical lymph node metastasis, lymph node dissection in regions II, III, IV, and Vb should also be performed (1). The ATA2015 version recommends that thyroid papillary carcinoma with lateral cervical lymph node metastasis should be considered as ipsilateral or bilateral CLND (2). However, 3%-30% of the patients with unilateral PTC still have occult lymph node metastasis in the contralateral central area (3), which significantly increases the risk of disease recurrence and metastasis. But whether contralateral CLND is needed for unilateral PTC with LLNM remains unclear, as this technique has been linked to an increased risk of recurrent laryngeal nerve injury and paranasal gland injury.

Currently, preoperative ultrasound can only identify half of the CLNs resulting from the presence of structures such as the thyroid gland, trachea, and sternum overlying the lymph nodes (4); as such, these images cannot serve as a guiding recommendation for surgical plans. This has led many surgeons to search for clinicopathological features which may serve as independent predictors for contralateral central lymph node metastasis (cCLNM). As shown in earlier studies, the occurrence of cCLNM in unilateral PTC may be linked to male sex, young age (<45 years), multifocality, location of the primary tumor (in the inferior region of the thyroid or not), tumor diameter >1 cm, capsular invasion, and ipsilateral (i)CLNM (3, 5–9). However, the risk factors of cCLNM in unilateral PTC patients with LLNM is still need to clarify. Therefore, in the present study, we analyzed the risk of cCLNM in uPTC with LLNM and investigated the optimal range of lymph node dissection by comparing the 5-year recurrence-free survival rates.

## 2 Methods

### 2.1 Patients and methods

We retrospectively reviewed the records of 888 patients pathologically confirmed as having unilateral PTC with LLNM. Thyroidectomy with neck dissection was performed on all patients at the Department of Head and Neck Surgery, Harbin Medical University Cancer Hospital from 2008 to 2022. The inclusion criteria were as follows: 1) thyroid carcinoma with LLNM suspected on preoperative ultrasound or imaging, 2) no prior record of thyroid surgery or head and neck radiotherapy, and 3) underwent total thyroidectomy + CLND + selective LLND. The exclusion criteria were as follows: 1) non-PTC (medullary/follicular/interstitial degeneration), 2) bilateral PTC confirmed by postoperative pathological examination, 3) no LLNM confirmed by postoperative pathology, 4) preoperative distant metastasis, and 5) incomplete clinical information or refusal to participate in the study.

Baseline clinicopathological information, including preoperative clinicopathological features, lymph node metastasis, and post-observation information, was collected for all patients. The surgeon collaboratively determined the extent of the surgical procedure by considering the established criteria and patient preferences. Lymph node dissection was performed in strict accordance with the CSCO guidelines for differentiated thyroid management. Recurrence was defined as any cytologically or pathologically confirmed lesion after surgery, or a disseminated metastatic condition identifiable on cross-sectional imaging or RAI scans. Follow-up care after surgery was adapted and personalized to address the likelihood of recurrence and efficacy of the initial therapy. Patients lost to follow-up were excluded from the final analysis.

The study protocol was approved by Ethics Committee of the Affiliated Cancer Hospital of Harbin Medical University, and all patients provided written informed consent before enrollment.

### 2.2 Propensity score matching analysis

**Figure 1:**
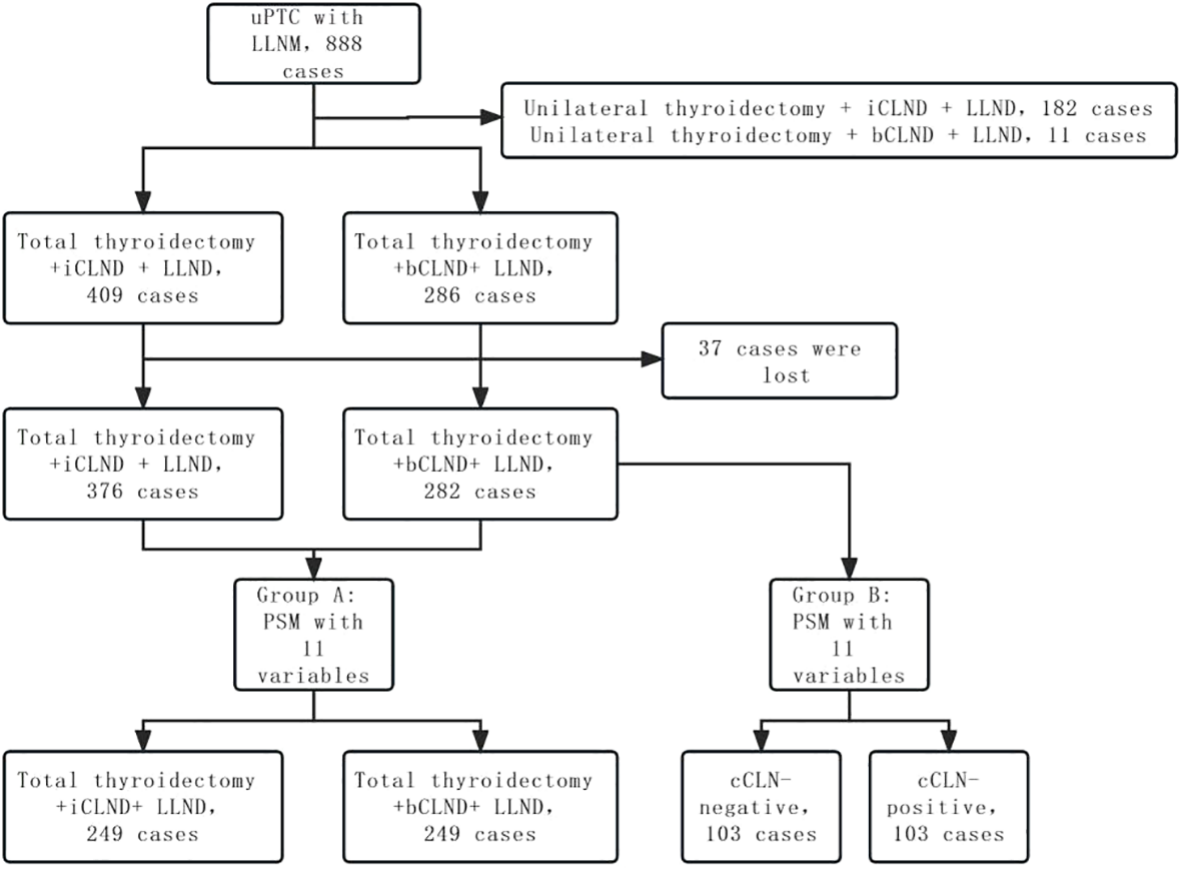
Flow chart showed matched grouping of patients with unilateral PTC with LLNM.

Propensity score matching (PSM) was performed to select for confounding factors. Eleven variables were included in the PSM: sex, age, multifocality, location of primary tumor, tumor diameter, capsular invasion, combined thyroid nodular goiter, combined Hashimoto thyroiditis, iCLNM and number of LLNM, and adjuvant iodine-131 treatment. Group A was obtained by PSM between patients undergoing total thyroidectomy + bCLND and those undergoing total thyroidectomy + iCLND. Patients who received total thyroidectomy + CLND underwent PSM according to whether there was cCLNM to obtain group B. The recurrence-free survival (RFS) rates of the two groups were compared (**Figure 1**).

### 2.3 Statistical analysis

Statistical analyses were performed using SPSS version 25.0. Receiver operating characteristic (ROC) curve analysis was employed to determine the threshold tumor diameter and number of LLNM to predict the risk of cCLNM. For baseline clinical characteristics, single-variable analysis was conducted using the test for continuous variables and the chi-square test for categorical variables. Thereafter, a prediction model was established by including the factors that showed significant results in the univariate analysis into multivariate Logistic regression equation, and evaluated by Hosmer-Lemeshow goodness-of-fit test. Recurrence-free survival (RFS) was assessed using Kaplan-Meier survival analysis, and the log-rank test was used for comparison. Statistical significance was set at *P*<0.05.

## 3 Results

### 3.1 Baseline clinicopathological features

A comprehensive sample of 888 patients with unilateral PTC and LLNM was analyzed in this study, including 286 patients who underwent total thyroidectomy + bCLND + selective LLND, 409 patients who underwent total thyroidectomy + iCLND + selective LLND, 182 patients who underwent unilateral thyroidectomy + iCLND + selective LLND, and 11 patients who underwent unilateral thyroidectomy + bCLND + selective LLND. Of the 695 patients who underwent total thyroidectomy + LLND, 71.5% (497/695) were women; the mean age was 39.7 (11-79) years; the mean maximum tumor diameter was 17.2 (3-89) mm; and 82% (571/695) had iCLNM. Among the patients who underwent total thyroidectomy + bCLND + selective LLND, 86.7% (248/286) had iCLNM and 52% (149/286) had cCLNM. Among patients who underwent total thyroidectomy + iCLND + selective LLND, 78.9% (323/409) had iCLNM.

### 3.2 Risk factors for cCLNM

**Figure 2:**
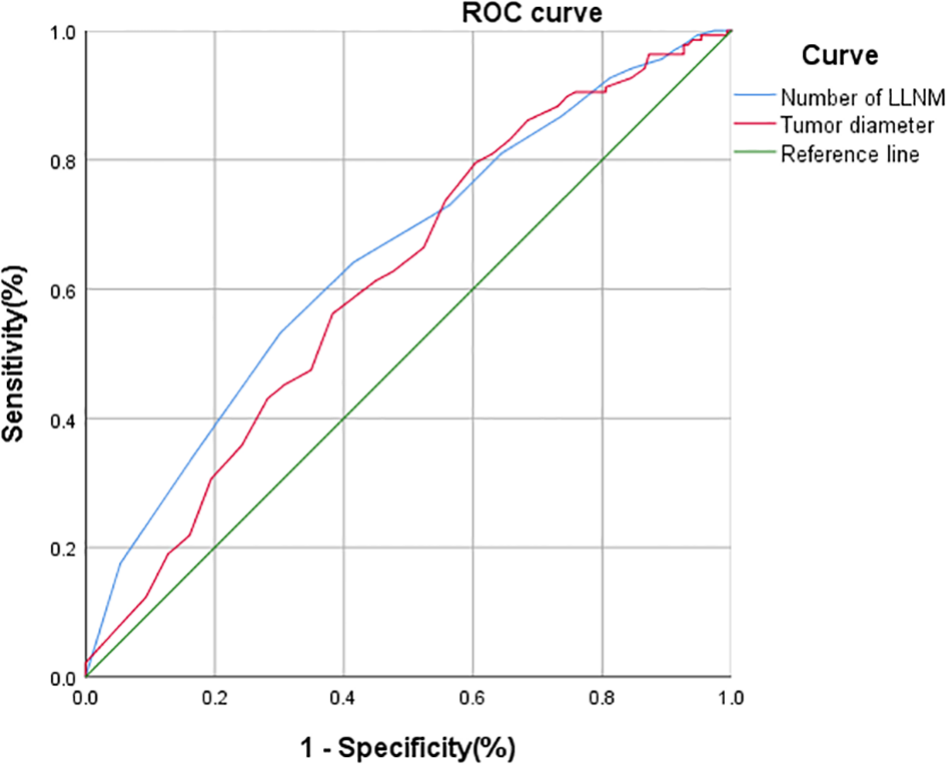
ROC analysis of tumor diameter, and the number of LLNM for cCLNM. Tumor diameter: AUC: 0.614 95%CI: 0.549~0.679; *P*=0.001. Number of LLNM: AUC: 0.650 95%CI: 0.587~0.714; *p <*0.001.

ROC curve analysis was performed in 286 patients who underwent bCLND to determine threshold values for tumor diameter and number of LLNM to predict cCLNM. The area under the curve (AUC) for tumor diameter from the ROC curve analysis was 0.614 (95% CI: 0.549-0.679; *P*=0.001), and the AUC for the number of LLNM was 0.650 (95% CI: 0.587-0.714; *P*<0.001). The analysis showed that a tumor diameter size of 20.5 mm and 3.5 for number of LLNM were effective predictors of cCLNM (**Figure 2**). Univariate analysis showed that combined thyroid nodular goiter, combined Hashimoto’s thyroiditis, tumor diameter >2 cm, iCLNM, and number of LLNM >3 were significantly associated with cCLNM (**Table 1**). Variables found to be significant in the single-variable analysis were incorporated into the multivariable logistic regression equation (**Table 2**). The results showed that the larger diameter of tumor, the higher risk of cCLNM (OR=2.11;95%Cl1.204-3.679;*P*=0.009). Meanwhile, the number of LLNM > 3(OR=2.02;95%Cl:1.201-3.396;*P*=0.008) and iCLNM(OR=3.966;95%Cl:1.69-9.309;*P*=0.002) also increased the risk of cCLNM. The calibration ability of the prediction model was evaluated by Hosmer-Lemeshow goodness-of-fit test(*X* ^2^ = 3.809,*P*=0.802 > 0.05). The results showed that there is no statistical difference between predicted value and actual observed value of the model. And the predictive model has good calibration ability.

**Table 1:** Results of univariate analysis to identify factors associated with cCLNM.

	cCLN-negative (n=137)	cCLN-positive (n=149)	Univariate analysis	
			*X* ^2^	*P*
Gender				
Male/Female	27/110	43/106	3.233	0.072
Age				
<45 years	102	118	0.904	0.342
≥45 years	35	31		
Hashimoto thyroiditis				
Yes	59	47	4.062	0.044
No	78	102		
Thyroid nodular goiter				
Yes	91	115	4.100	0.043
No	46	34		
Multifocality				
Single	84	93	0.037	0.848
Multiple	53	56		
Tumor diameter				
≤2cm	109	90	12.378	<0.001
>2cm	28	59		
Location of the primary tumor				
The inferior region	40	58	2.999	0.083
Location other than the inferior region	97	91		
Capsular invasion				
Yes	83	101	1.613	0.204
No	54	48		
iCLNM				
Yes	107	141	16.924	<0.001
No	30	8		
Number of LLNM				
≤3	73	45	15.692	<0.001
>3	64	104		

cCLN, contralateral central lymph node; cCLNM, contralateral central lymph node metastasis; iCLNM, ipsilateral central lymph node metastasis; LLNM, lateral lymph node metastasis.

**Table 2:** Results of multivariate logistic regression analysis of cCLNM.

	B(SE)	P	OR	OR 95%Cl	
				Lower	Upper
Thyroid nodular goiter	0.456 (0.294)	0.121	1.577	0.886	2.805
Hashimoto thyroiditis	-0.421 (0.272)	0.121	0.656	0.386	1.118
Tumor diameter>2cm	0.747 (0.286)	0.009	2.11	1.204	3.697
iCLNM	1.378 (0.453)	0.002	3.966	1.69	9.309
Number of LLNM>3	0.703 (0.265)	0.008	2.02	1.201	3.396
Constant	-1.934 (0.498)	0	0.145		

iCLNM, ipsilateral central lymph node metastasis; LLNM, lateral lymph node metastasis.

### 3.3 PSM

Propensity score matching (PSM) was performed to produce matched groups. A total of 249 pairs of patients who underwent total thyroidectomy + iCLND and total thyroidectomy + bCLND were matched to obtain group A, and 103 pairs of cCLN-negative and cCLN-positive patients were matched to obtain group B (**Table 3**). In matched group A, no significant statistical disparity was detected in the dose of postoperative iodine-131 adjuvant therapy between the iCLND and bCLND groups (2912.45 ± 3253.182 *vs*. 3402.81 ± 3566.244, *P*=0.110). In matched group B, there was no statistical difference in the postoperative iodine-131 adjuvant therapy dose between patients in the cCLN-negative and cCLN-positive groups (3412.62 ± 3051.508 *vs*. 3807.77 ± 4061.626, *P*=0.431). Further, there were no statistically significant differences in sex, age, multifocality, location of primary tumor, tumor diameter, capsular invasion, combined thyroid nodular goiter, combined Hashimoto thyroiditis, iCLNM, or number of LLNM between the two matched groups (*p* > 0.05). In matched group A, the median follow-up times for patients in the iCLND and bCLND groups were 79 months (95% Cl 72.866-81.134, mean 63 months) and 52 months (95% Cl 38.012-65.988, mean 48 months), respectively. In matched group B, the median follow-up times for patients in the cCLN-negative and cCLN-positive groups were 53 months (95% Cl, 42.293–63.707, mean 44.8 months) and 55 months (95% Cl, 50.683–59.317, mean 51.4 months), respectively.

**Table 3:** Clinicopathological characteristics of the two matched groups after PSM.

	Group A				Group B			
	iCLND (n=249)	bCLND (n=249)	*X* ^2^/t	*P*	cCLN-negative (n=103)	cCLN-positive (n=103)	*X* ^2^/t	*P*
Gender								
M/F	70/179	67/182	0.091	0.763	21/82	30/73	2.111	0.146
Age								
<45 years	177	186	0.823	0.364	78	81	0.248	0.618
≥45 years	72	63			25	22		
Hashimoto thyroiditis								
Yes	74	82	0.597	0.440	43	38	0.509	0.476
No	175	167			60	65		
Thyroid nodular goiter								
Yes	194	191	0.103	0.748	76	77	0.025	0.873
No	55	58			27	26		
Multifocality								
Single	160	158	0.035	0.852	67	66	0.021	0.884
Multiple	89	91			36	37		
Tumor diameter								
≤2cm	168	173	0.602	0.438	76	71	0.594	0.441
>2cm	81	73			27	32		
Location of primary tumor								
The inferior region	91	84	0.432	0.511	35	39	0.337	0.561
Location other than the inferior region	158	165			68	64		
Capsular invasion								
Yes	136	156	3.312	0.069	61	66	0.512	0.474
No	113	93			42	37		
iCLNM								
Yes	209	214	0.392	0.531	94	95	0.064	0.800
No	40	35			9	8		
Number of LLNM								
≤3	97	106	0.674	0.412	46	39	0.981	0.322
>3	152	143			57	64		
Iodine-131 adjuvant treatment(MBq)	2912.45 ± 3253.182	3402.81 ± 3566.244	1.6 03	0.1 10	3412.62 ± 3051.508	3807.77 ± 4061.626	0.7 89	0.4 31

iCLND, ipsilateral central lymph node dissection; bCLND, bilateral central lymph node dissection; cCLN, contralateral central lymph node; iCLNM, ipsilateral central lymph node metastasis; LLNM, lateral lymph node metastasis.

### 3.4 Postoperative recurrence

**Figure 3:**
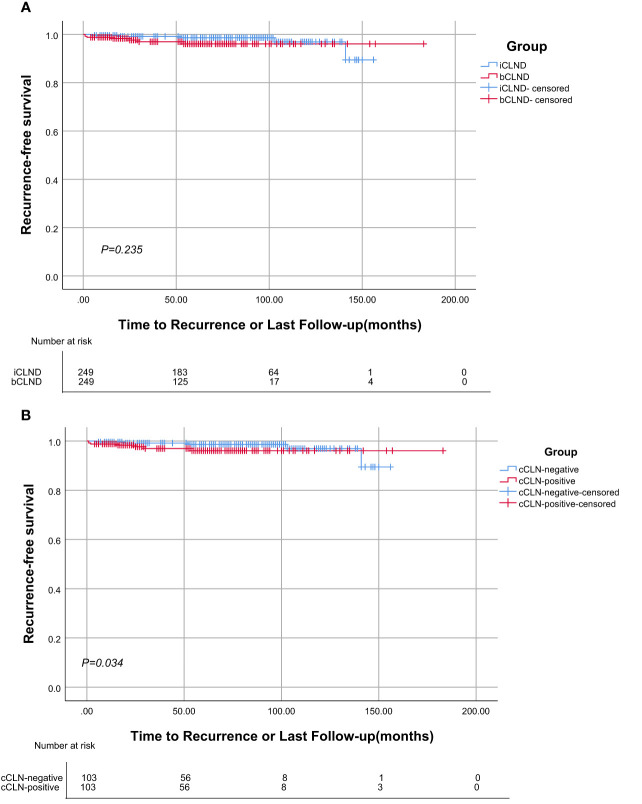
Kaplan-Meier analysis of Recurrence-free survival. **(A)** There was no statistically significant difference in recurrence-free survival curve between the iCLND and bCLND groups (*P*=0.235). **(B)** Statistical differences in the Kaplan-Meier recurrence-free survival curves between the cCLN-negative and cCLN-positive groups (*P*=0.034).

In matched group A, five cases relapsed in the iCLND group, with a 5-year RFS rate of 98.6%, while seven cases relapsed in the bCLND group with a 5-year RFS rate of 96.1%; there were no significant differences between the two groups (*X* ^2^ = 1.409, *P*=0.235) (**Figure 3A**). In matched group B, there was one recurrence in the cCLN-negative group with a 5-year RFS of 98.5% and seven recurrences in the cCLN-positive group with a 5-year RFS of 91.1%, with a statistically significant difference between the two groups (*X* ^2^ = 4.489, *P*=0.034) (**Figure 3B**).

The rates of reoperation for recurrence were similar in the iCLND and bCLND groups (1.2%, 3/249 *vs*. 2.8%, 7/249; *P*=0.559); however, difference in reoperation rates was observed between the cCLN-negative and cCLN-positive groups (1%, 1/103 *vs*. 6.8%, 7/103; *P*=0.030). We summarized the incidence and details of reoperation (**Table 4**). Two patients with distant lung metastases were subsequently treated with adjuvant iodine-131 in the iCLND group, and one patient presented with dedifferentiated thyroid carcinoma during reoperation in the cCLN-positive group; the rest were PTC.

**Table 4:** Incidence and details of reoperation in Groups A and B.

	Group A		Group B	
	iCLND (n=249)	bCLND(n=249)	cCLN-negative (n=103)	cCLN-positive (n=103)
Incidence of reoperation				
Number of surgeries	3(1.2%)	7(2.8%)	1(1%)	7(6.8%)
Distance metastasis	2	0	0	0
Details of reoperation				
iCLN	1	1	0	2
Ipsilateral LLN	1	3	1	2
cCLN	1	0	0	0
Contralateral LLN	0	3	0	3

iCLND, ipsilateral central lymph node dissection; bCLND, bilateral central lymph node dissection; cCLN, contralateral central lymph node; iCLN, ipsilateral central lymph node; LLN, lateral lymph node.

## 4 Discussion

As the surgical treatment strategy for PTC has become more refined, the optimal range of lymph node dissection in unilateral PTC patients with cCLNM has received increasing attention. Prior studies have shown that complete resection of the CLN can clear potential cCLNM (10, 11), while intraoperative exploration may increase the chance of damage to the laryngeal recurrent nerve and parathyroid glands, which, although highly unlikely, will cause serious complications to patients once it occurs. Therefore, it is important to investigate the independent risk factors of cCLNM. Although many studies have examined this issue, the majority did not examine patients with LLNM. In this study, we included patients with unilateral PTC with LLNM and performed two subgroup analyses using PSM. No significant statistical variance was observed between matched groups A and B in terms of sex, age, multifocality, location of primary tumor, tumor diameter, capsular invasion, combined thyroid nodular goiter, combined Hashimoto thyroiditis, iCLNM and number of LLNM, or postoperative iodine-131 adjuvant therapy dose. Further, we analyzed the effects of different lymph node dissection ranges and lymph node metastasis extents on the 5-year RFS after surgery, to explore the optimal extent of lymph node dissection.

Preoperative ultrasonography often cannot detect all CLNs because of low coverage of the thyroid, trachea, and sternum (4). In our study, the rate of iCLNM was 82% (571/695), which corroborates earlier study results (9, 12–14); while the rate of cCLNM was as high as 52% (149/286), which is higher than the rates of 3%-30% observed in previous studies (3). This discrepancy may be explained by the fact that all patients enrolled in this study had unilateral PTC with LLNM. This suggests that cCLNM should be considered in unilateral PTC with LLNM.

Thyroidectomy + CLND + selective LLND is the mainstream surgical strategy for PTC with LLNM. However, prophylactic CLND is controversial owing to its special location (15, 16), as it may cause serious complications in patients, including hoarseness and severe calcium deficiency. However, several prior studies have indicated that prophylactic CLND significantly reduces the risk of local recurrence (17, 18). Our study also found that in patients who underwent bCLND, the 5-year RFS exhibited superior outcomes in the cCLN-negative group than in the cCLN-positive group, and the risk of local recurrence was greatly elevated in the presence of cCLNM.

As a primarily metastasizes, lymphatic metastasis of PTC generally occurs via the following mechanism: first, cancer cells metastasize to the lymph nodes in the central region, with a metastasis rate as high as 82% (19); cells then spread to lateral cervical lymph nodes (primarily IV, III, and II); and finally, cells spread to form distant metastases in organs such as the lungs, bones, and liver. But some studies also have reported leapfrog metastases without metastasis to iCLN (20). Therefore, adequate lymph node dissection is crucial to reduce the postoperative recurrence rate. However, it should be noted that local recurrence due to incomplete lymph node dissection accounted for a significant proportion of patients with recurrence in this study, with four (40%, 4/10) cCLN or contralateral lateral lymph node recurrences in matched group A and three (37.5%, 3/8) contralateral lateral lymph node recurrences in matched group B. Despite the lack of a statistically significant difference in the 5-year RFS rate between the bCLND and iCLND groups, this may be due to the limitation of CLN ultrasonography, which cannot detect all local recurrences. Furthermore, the absence of prophylactic cCLND may also lead to missing lesions, which are prone to “insufficient staging” caused by incomplete lymph node dissection, resulting in inaccurate diagnosis (21, 22). In addition, missed lesions further increase the possibility of mediastinal lymph node metastasis, thus increasing the possibility of distant metastasis, such as to the lung (21–23). Even worse is the presence of missed lesions allows for the possibility of continued disease progression, as in the case of dedifferentiated thyroid cancer resulting from PTC dedifferentiation in our study, which, despite its low likelihood, would also present an extremely poor prognosis for the patient. This suggests that not all unilateral PTC with LLNM require cCLND; however, when there is a high risk of cCLNM, cCLND is necessary.

Using ROC curve analysis, we found that a tumor diameter size of 20.5 mm and 3.5 for number of LLNM were the most sensitive thresholds for predicting cCLNM. Using univariate and multifactorial logistic regression equation, we identified tumor diameter >2 cm, iCLNM, and number of LLNM >3 as independent risk factors of cCLNM. Unlike previous studies, our study identified a tumor diameter size of 20.5 mm as a predictive factor, which is larger than the tumor diameter >1 cm reported in previous studies (5, 6, 8). This may be because our study included only unilateral PTC with LLNM. Thus, these results suggest that individuals with unilateral PTC should be evaluated for the suitability of bCLND with tumor diameter >2 cm, iCLNM, and number of LLNM >3, while patients should undergo only iCLND if there are no such risk factors.

Our study had some limitations. First, this was a retrospective study, and although the PSM method was used to balance the differences in baseline characteristics, the clinical data was limited. Second, the single-center design of this study may have introduced selection bias. As such, future multi-center randomized controlled trials are needed to verify and support the conclusions of the study. Therefore, in future studies, we will extend the follow-up time, and include more patients and research factors to further explore and verify this issue.

## 5 Conclusion

In this retrospective cohort study, we found that in unilateral PTC with LLNM, primary tumor diameter > 2 cm, iCLNM, and number of LLNM >3 were independent risk factors for cCLNM. Our study also found that not all cases of unilateral PTC with LLNM require prophylactic cCLND. We propose that prophylactic cCLND is only required in patients with the above independent risk factors, while patients lacking these factors require only iCLND.

## Data availability statement

The raw data supporting the conclusions of this article will be made available by the authors, without undue reservation.

## Ethics statement

The studies involving human participants were reviewed and approved by Ethics Committee of Harbin Medical University Cancer Hospital, Harbin Medical University. The patients/participants provided their written informed consent to participate in this study.

## Author contributions

LZ: Conceptualization, Data curation, Formal analysis, Investigation, Writing – original draft, Writing – review & editing. ZD: Conceptualization, Resources, Writing – original draft. JH: Conceptualization, Data curation, Writing – review & editing. WB: Data curation, Validation, Writing – review & editing. CN: Conceptualization, Funding acquisition, Project administration, Writing – review & editing.
